# Supplementary material for: Aging Promotes Spontaneous Liver Injury: Insights from Metabolic, Inflammatory, and Fibrotic Pathways in C57BL/6 Mice
Source: Biomolecules. 2025 Dec 11;15(12):1727. doi: 10.3390/biom15121727 (PMC12730500; doi:10.3390/biom15121727)
Supplement: Supplementary file 1 [file biomolecules-15-01727-s001.zip › biomolecules-3996050 Supplementary File - Western blot.pdf]

Figure 4

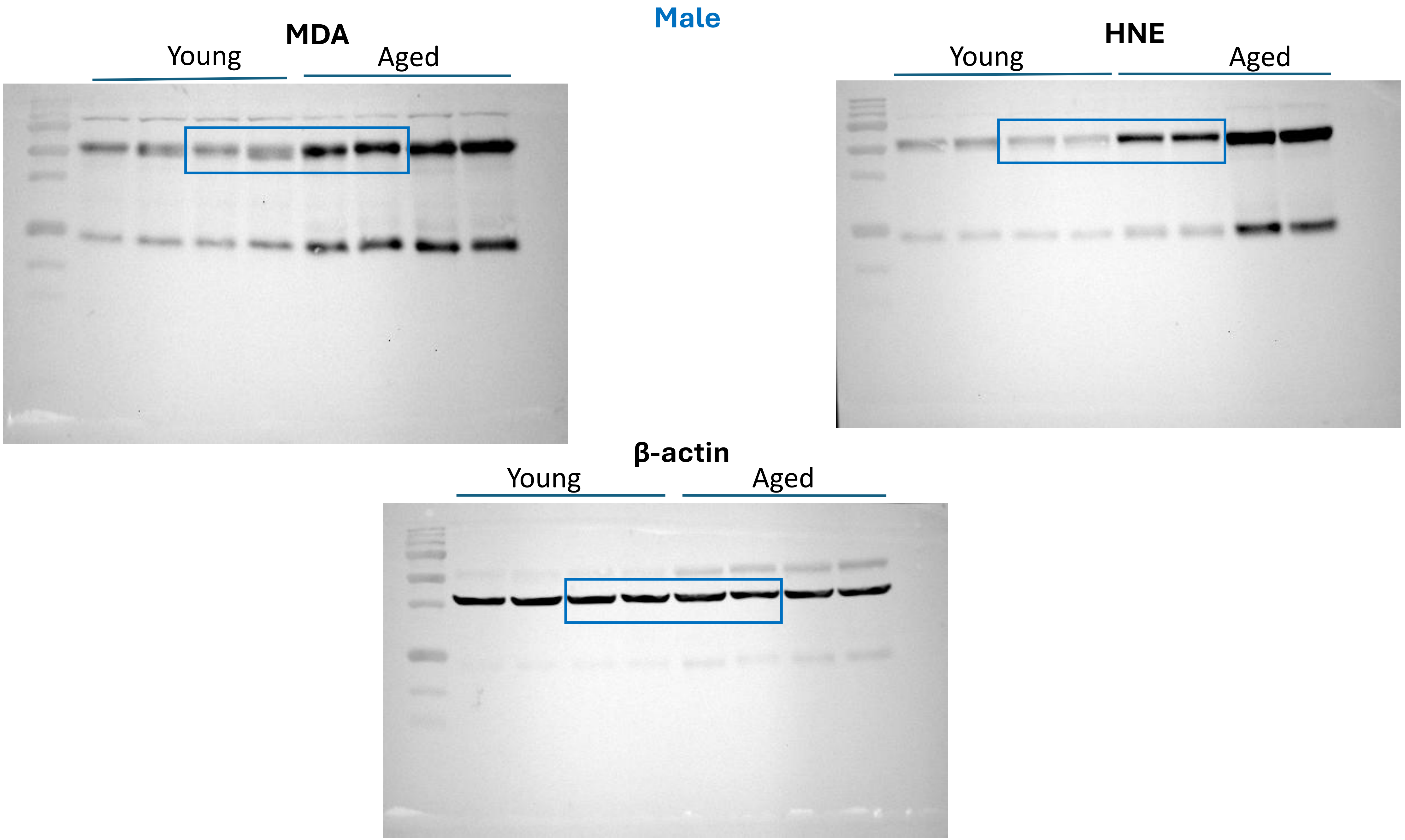

Figure 5

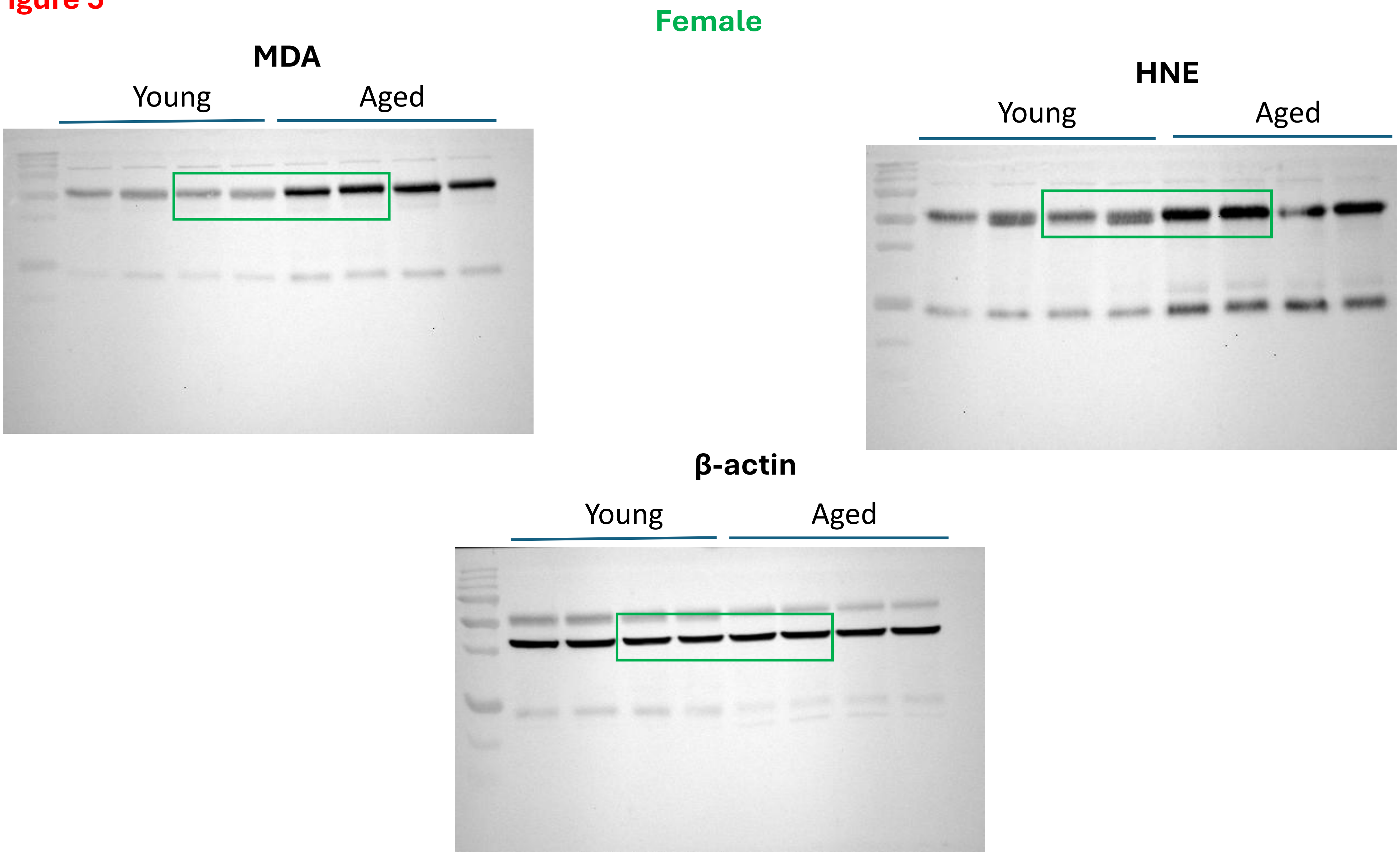

Figure 8

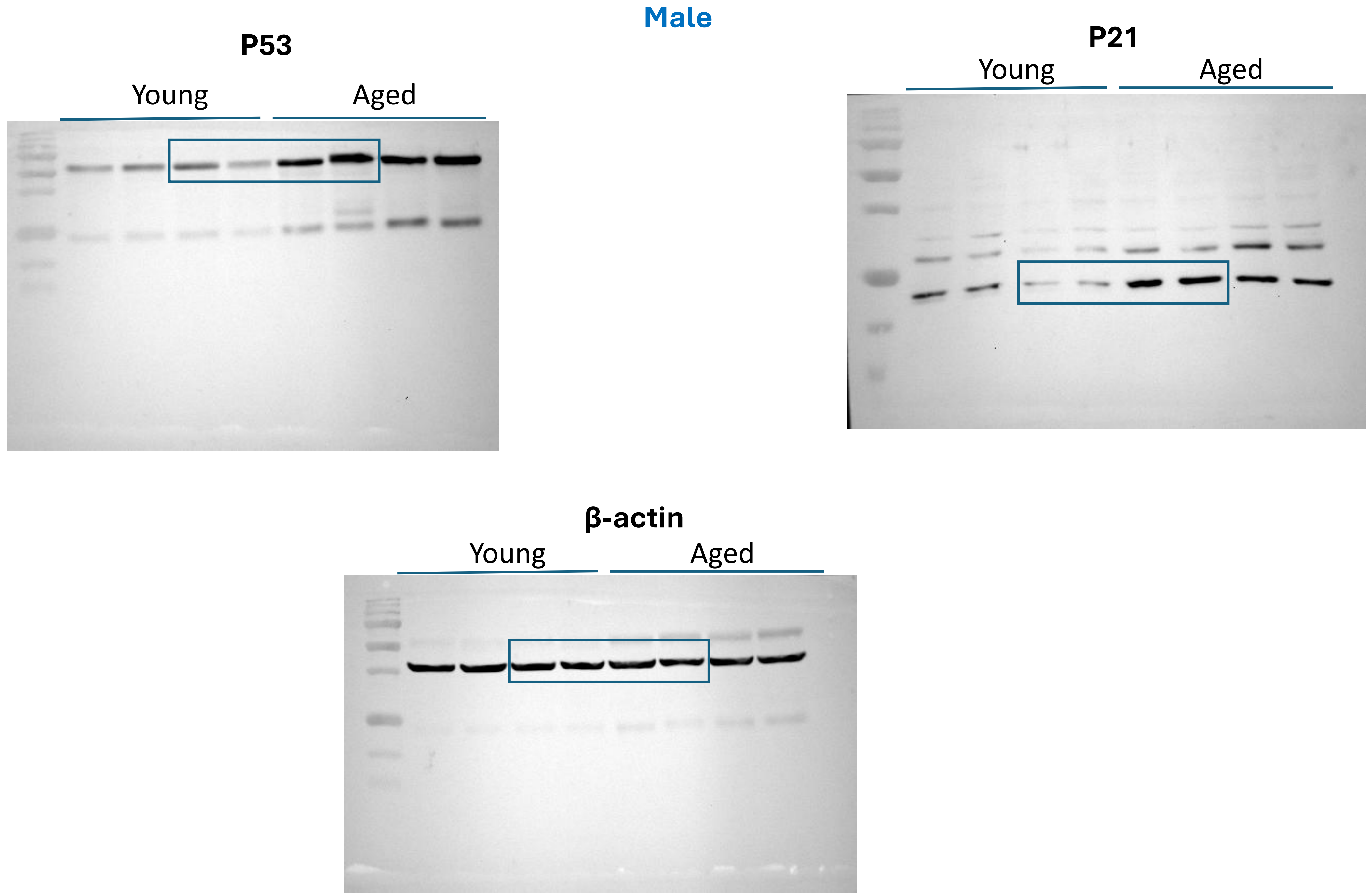

Figure 9

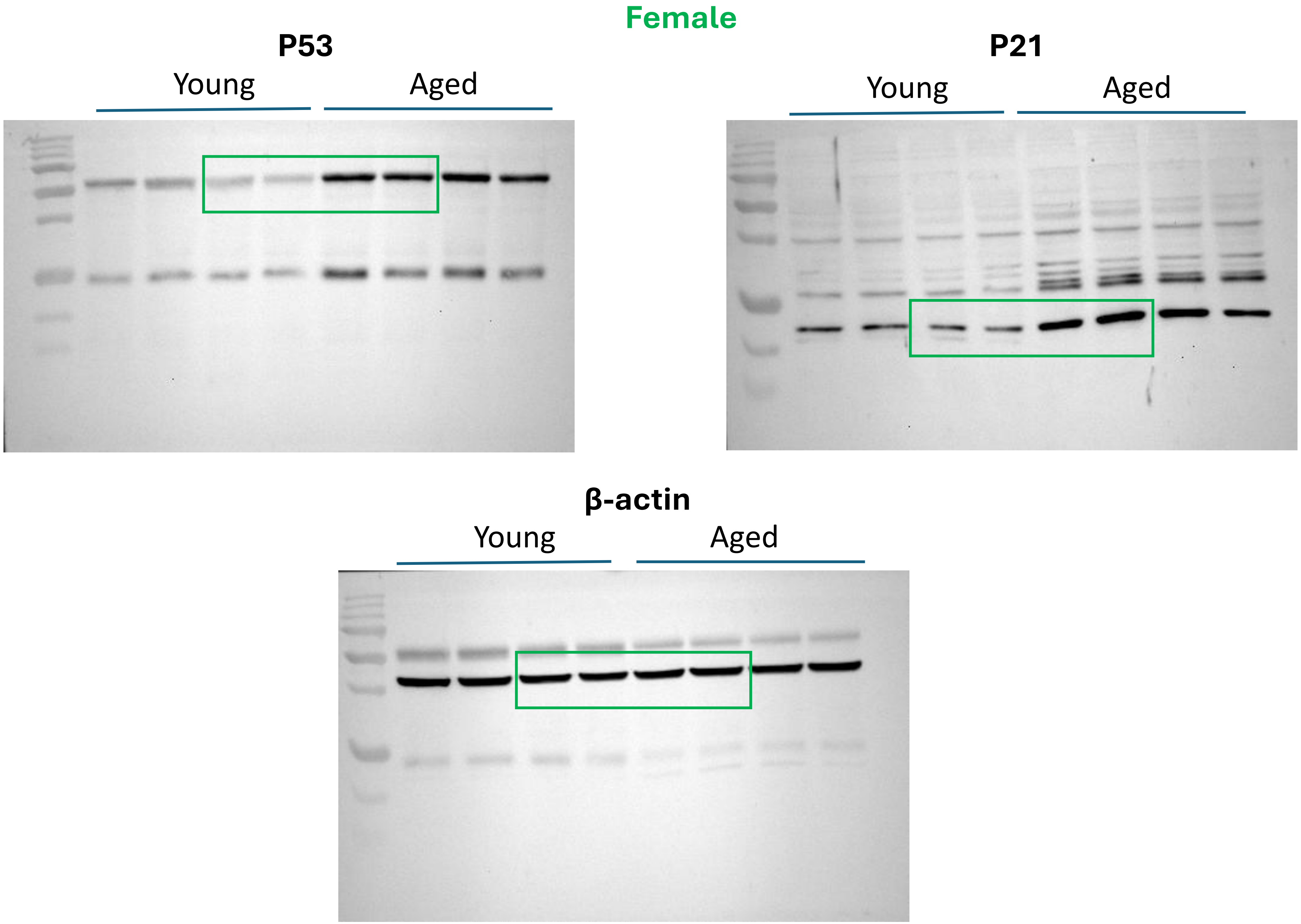

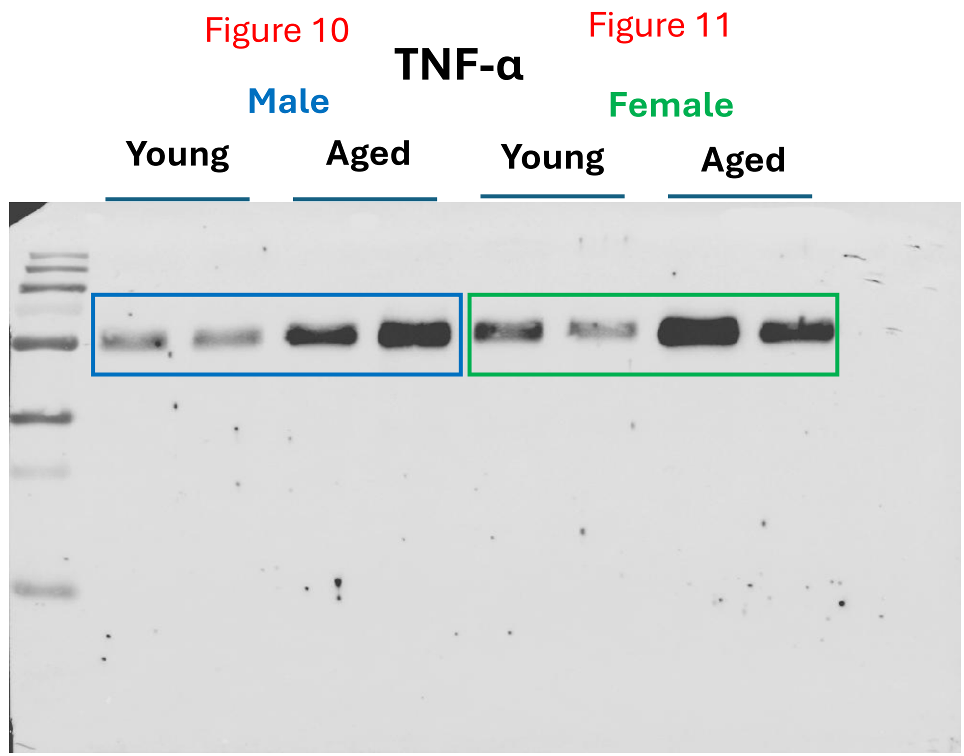
Figure 10
TNF-α
Figure 11
Male
Female
Young
Aged
Young
Aged

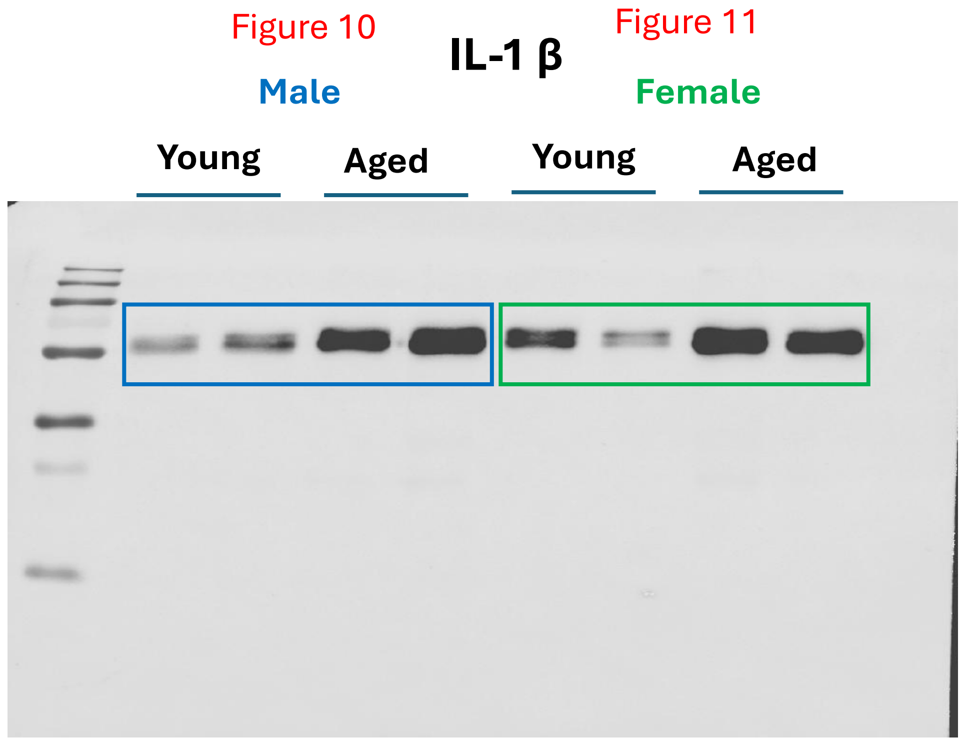
Figure 10
IL-1 β
Figure 11
Male
Female
Young
Aged
Young
Aged

Figure 10

CD-68

Male

Young Aged

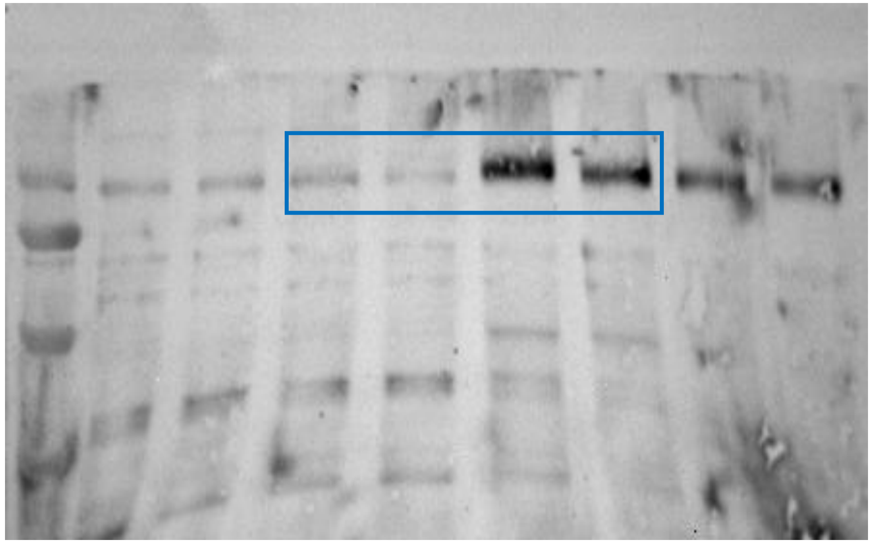

Figure 11

CD-68

Female

Young Aged

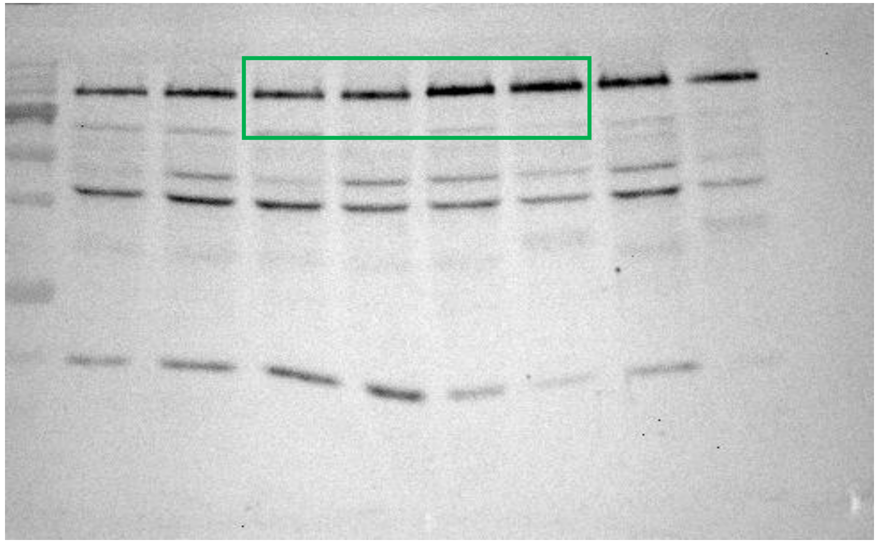

Myloperoxidase (MPO)

Figure 10

Male

Young Aged

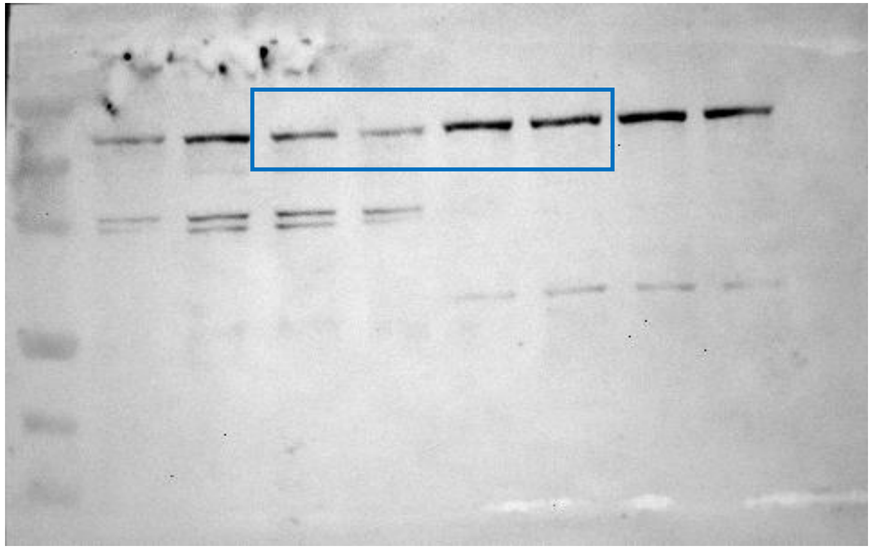

Myloperoxidase (MPO)

Figure 11

Female

Young Aged

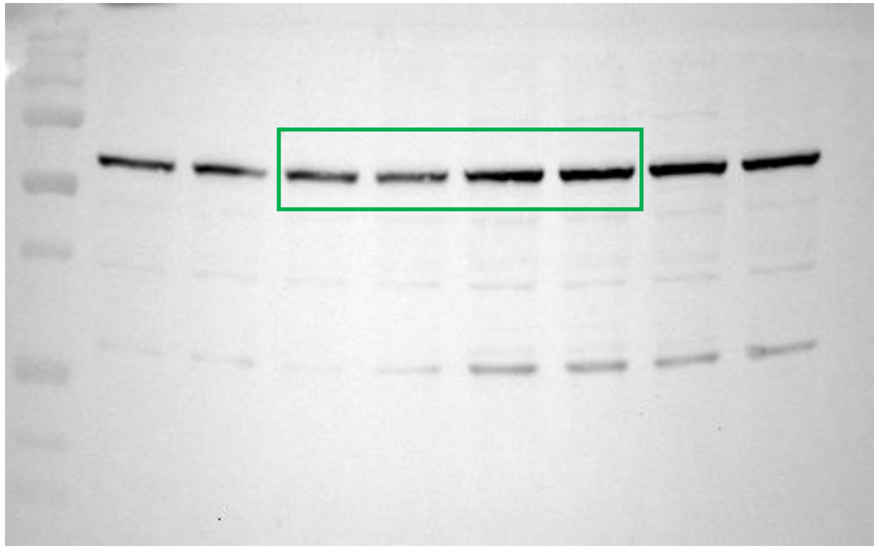

Figure 10

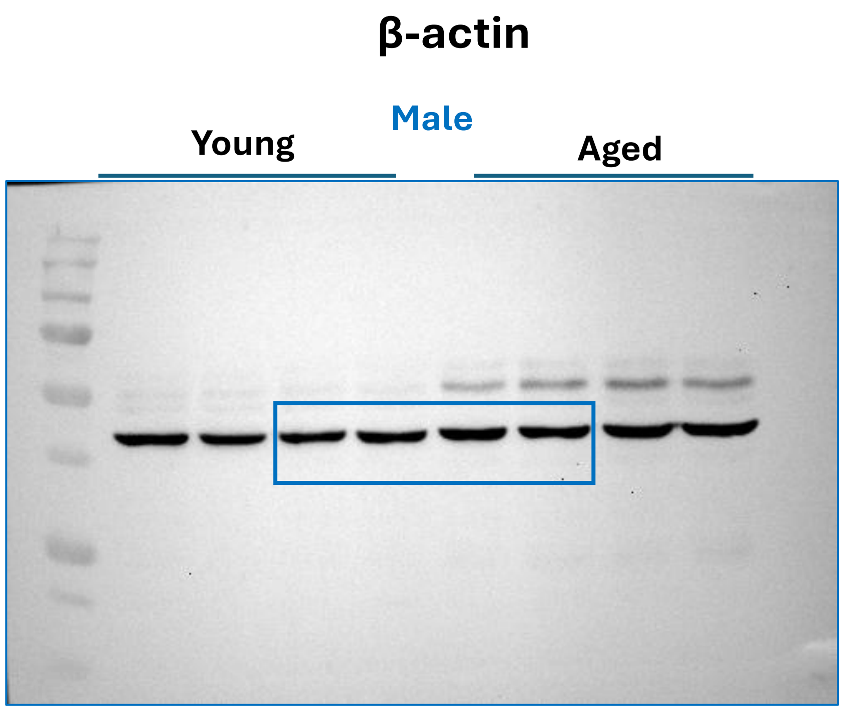

Figure 11

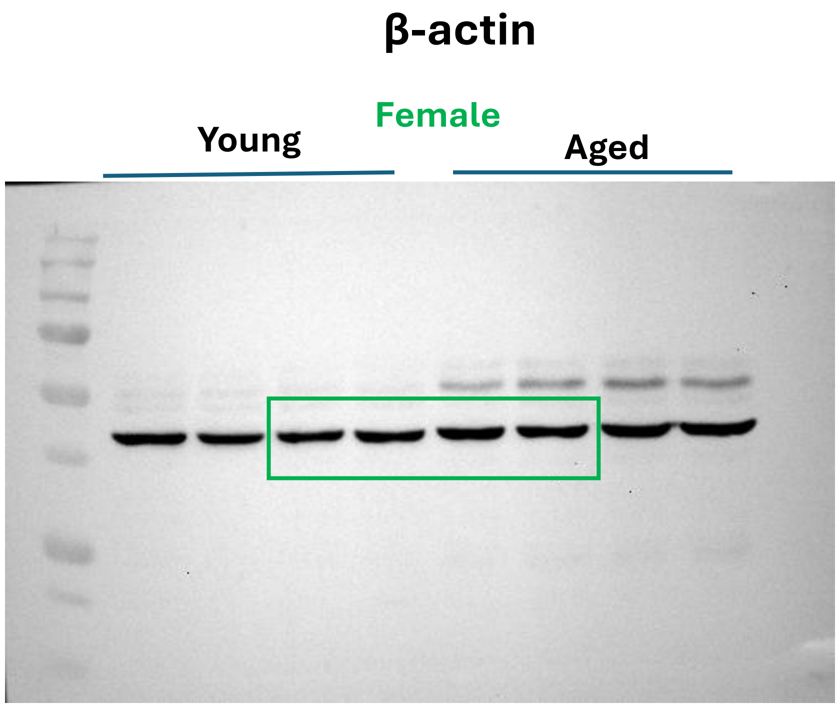

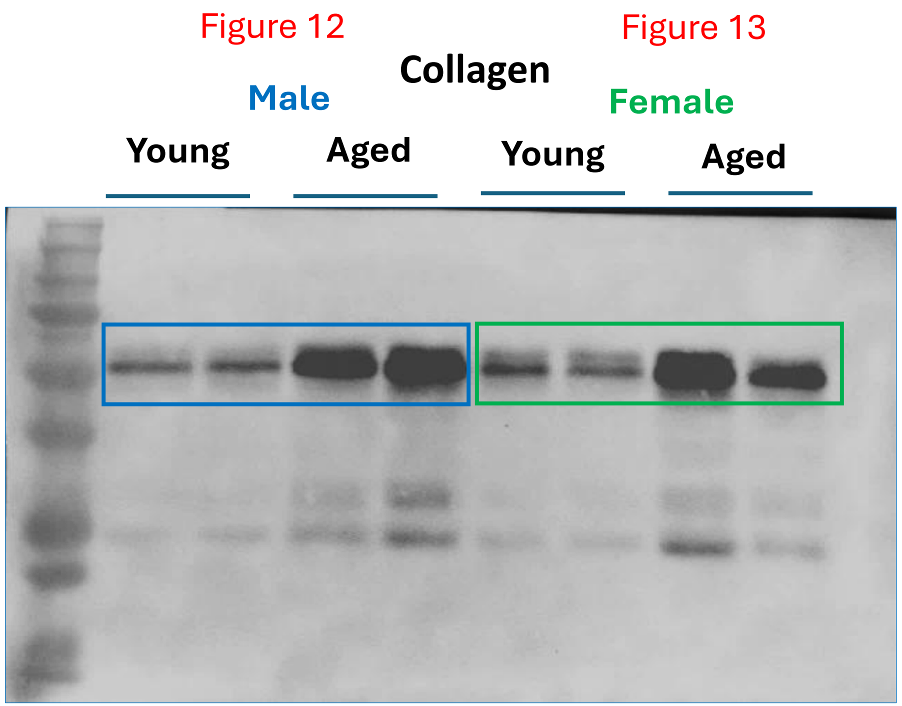
Figure 12
Collagen
Figure 13
Male
Female
Young
Aged
Young
Aged

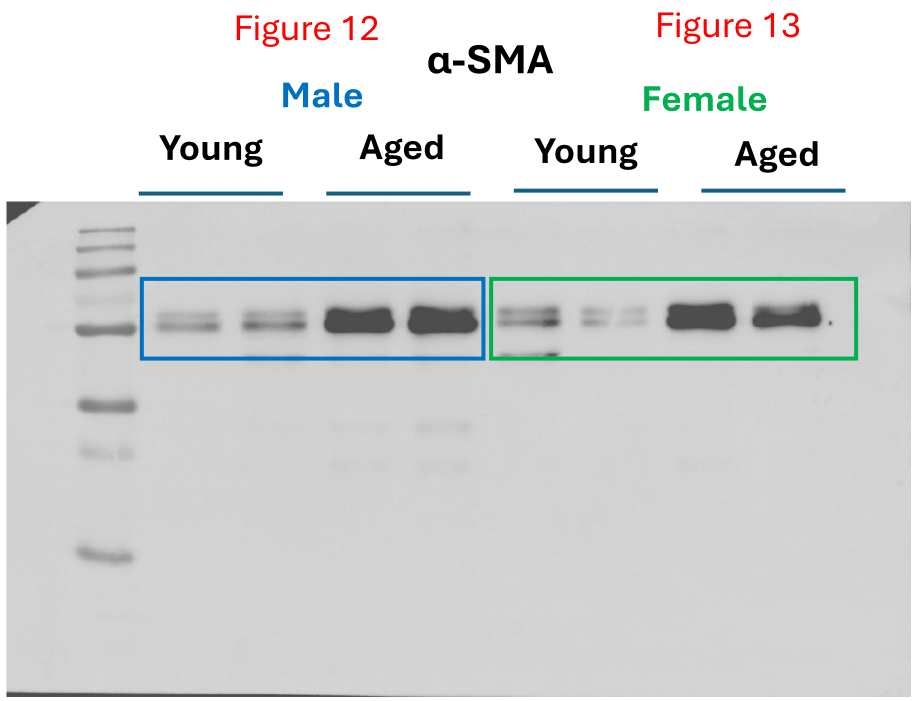
Figure 12
α-SMA
Figure 13
Male
Female
Young
Aged
Young
Aged

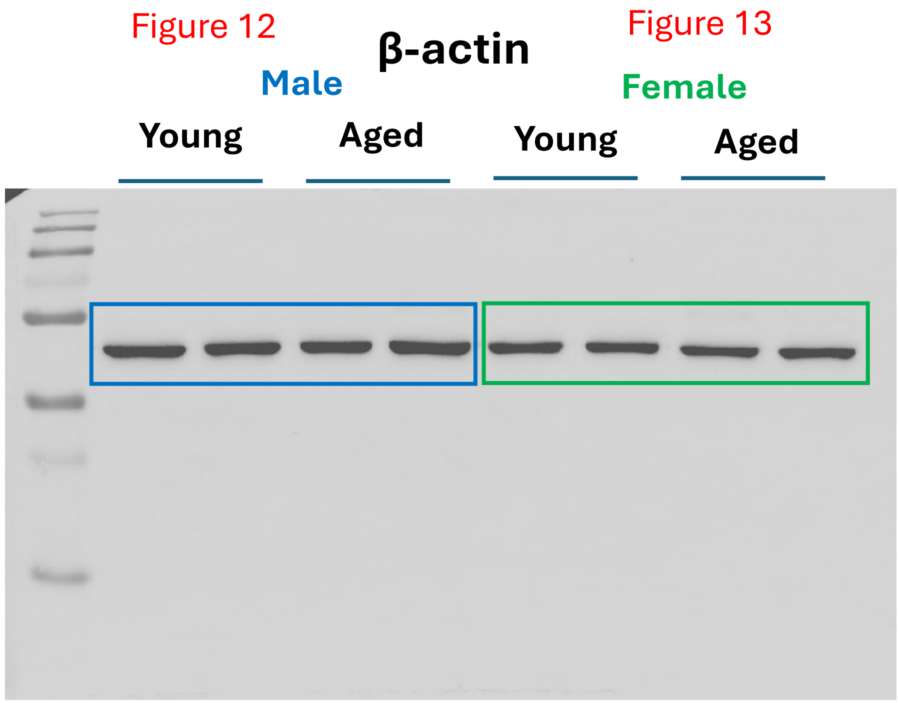
Figure 12
β-actin
Figure 13
Male
Female
Young
Aged
Young
Aged
