# Supplementary material for: Aging Promotes Spontaneous Liver Injury: Insights from Metabolic, Inflammatory, and Fibrotic Pathways in C57BL/6 Mice
Source: Biomolecules. 2025 Dec 11;15(12):1727. doi: 10.3390/biom15121727 (PMC12730500; doi:10.3390/biom15121727)
Supplement: Supplementary file 1 [file biomolecules-15-01727-s001.zip › biomolecules-3996050-Supplementary materials.pdf]

Supplementary information

Aging Promotes Spontaneous Liver Injury: Insights from Metabolic, Inflammatory, and Fibrotic Pathways in C57BL/6 Mice

Poonam Sagar [1,2,†], Sathish Kumar Perumal [1,2,†], Ramachandran Rajamanickam [1,2], Ramesh Bellamkonda [1,3], Sundararajan Mahalingam [1,3], Natalia A. Osna [4], Karuna Rasineni [1,3,*] and Kusum K. Kharbanda [1,2,3,*]

[1] Research Service, Veterans Affairs Nebraska-Western Iowa Health Care System, Omaha, NE 68105, USA; poonam181995@gmail.com (P.S.); sperumal@unmc.edu (S.K.P.); rrajamanickam@unmc.edu (R.R.); rbellamkonda@unmc.edu (R.B.); smahalingam@unmc.edu (S.M.)

[2] Department of Internal Medicine, University of Nebraska Medical Center, Omaha, NE 68198, USA

[3] Department of Biochemistry & Molecular Biology, University of Nebraska Medical Center, Omaha, NE 68198, USA

[4] Department of Pharmacology and Experimental Neuroscience, University of Nebraska Medical Center, Omaha, NE 68198, USA; nosna@unmc.edu

* Correspondence: karuna.rasineni@unmc.edu (K.R.); kkharbanda@unmc.edu (K.K.K.)

† These authors contributed equally to this work.

Karuna Rasineni, Ph.D., Department of Biochemistry and Molecular Biology, University of Nebraska Medical Center, Omaha, NE 68198-5870, USA. Tel.: +1-402-995-3752; Fax: +1-402-995-4600. E-mail: Karuna.rasineni@unmc.edu

Kusum K. Kharbanda, Ph.D., Veterans Affairs Nebraska-Western Iowa Health Care System, Research Service (151), 4101 Woolworth Avenue, Omaha, Nebraska, 68105, USA. Tel.: +1-402-995-3752; Fax: +1-402-995-4600. E-mail: Kkharbanda@unmc.edu

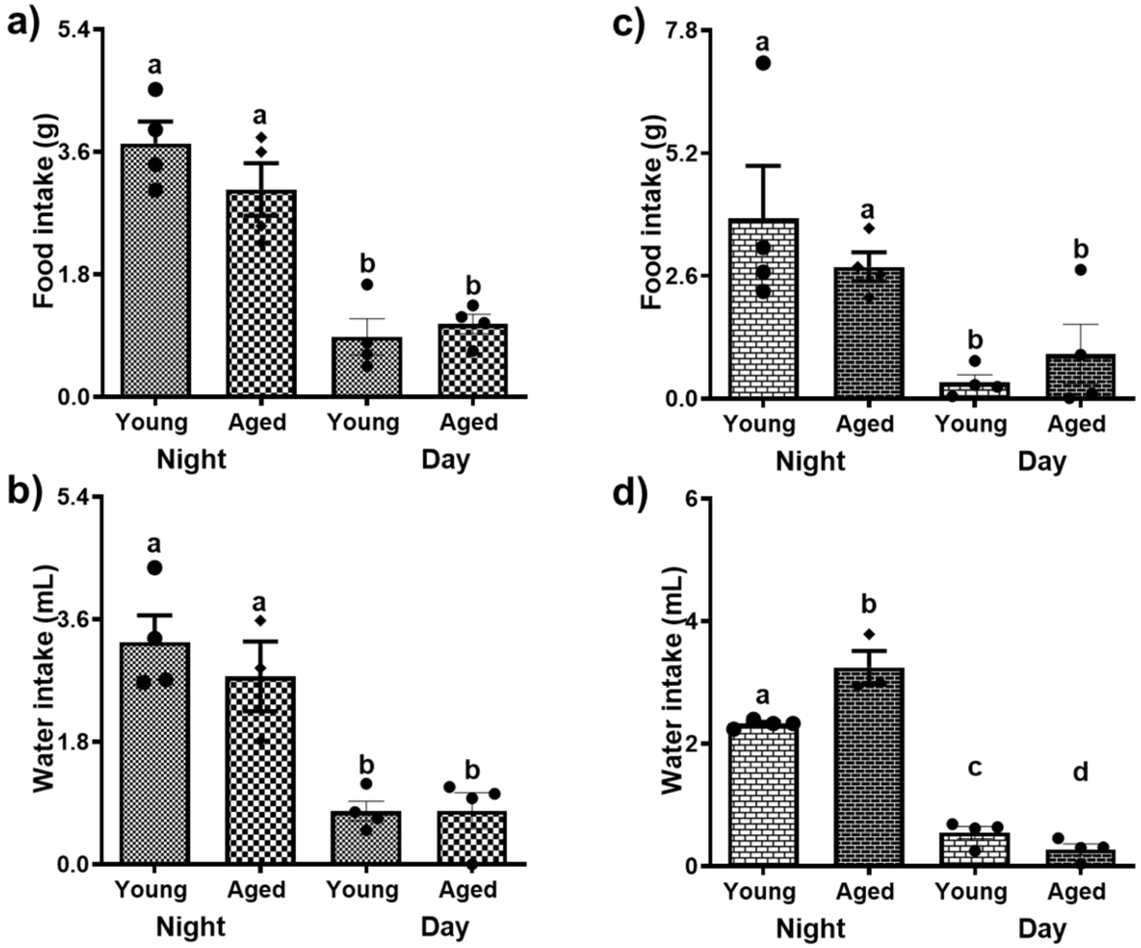

Figure S1. Sustenance parameters in young and aged male (a, b) and female (c, d) mice during the entire day. (a, c) Food and (b, d) water intake. Values are mean ± SEM (n = 5); values not sharing a common letter significantly differ from each other at $p \leq 0.05$.

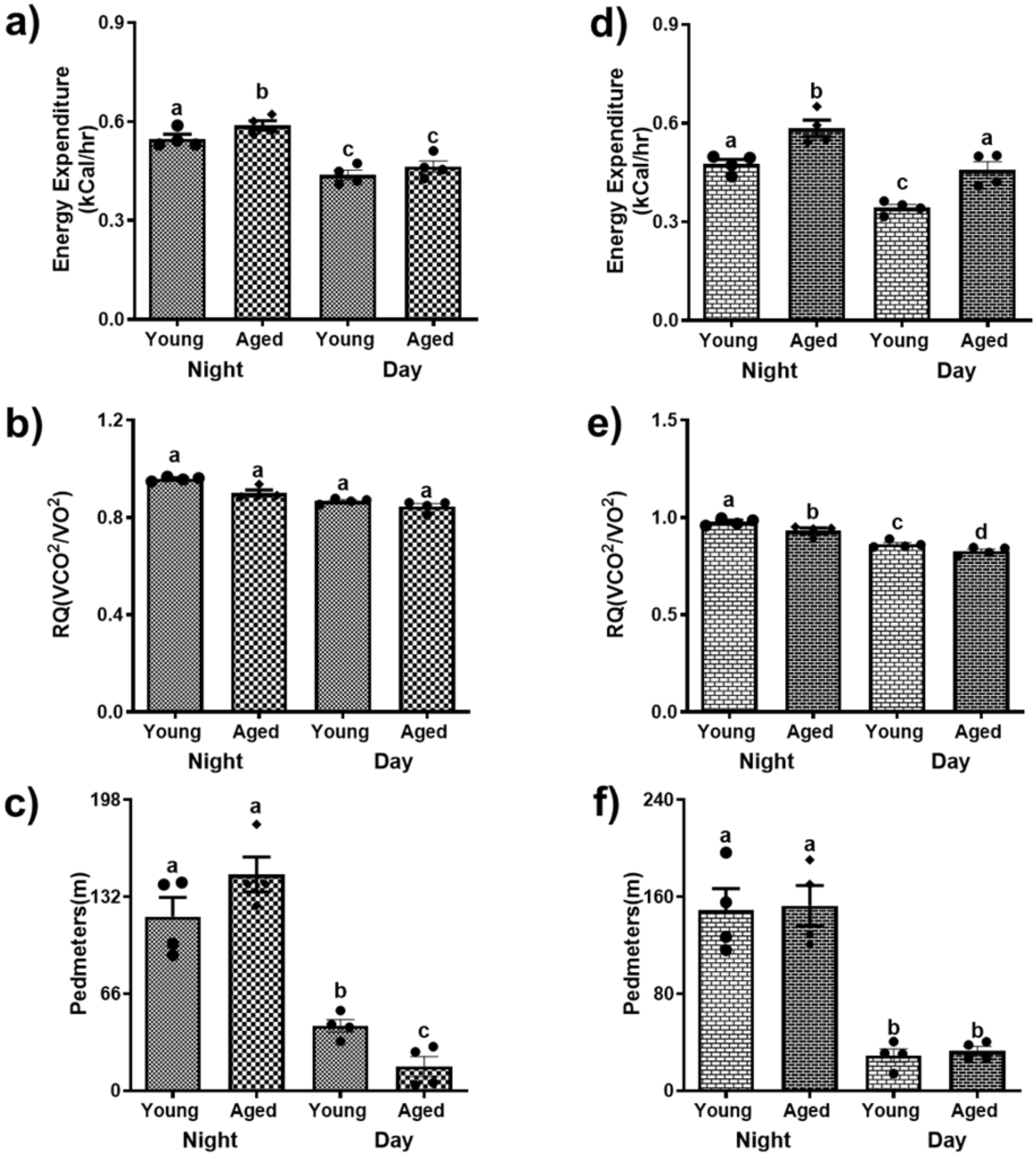

Figure S2. Metabolic parameters in young and aged male (a-c) and female (d-e) mice during the entire day. (a, d) Energy expenditure, (b, e) Respiratory quotient (RQ) profile, (c, f) pedometers (i.e., ambulatory locomotion of 1cm/second or above within the x,y,z beam-break system). Values are mean ± SEM (n = 5); values not sharing a common letter significantly differ from each other at $p \leq 0.05$.

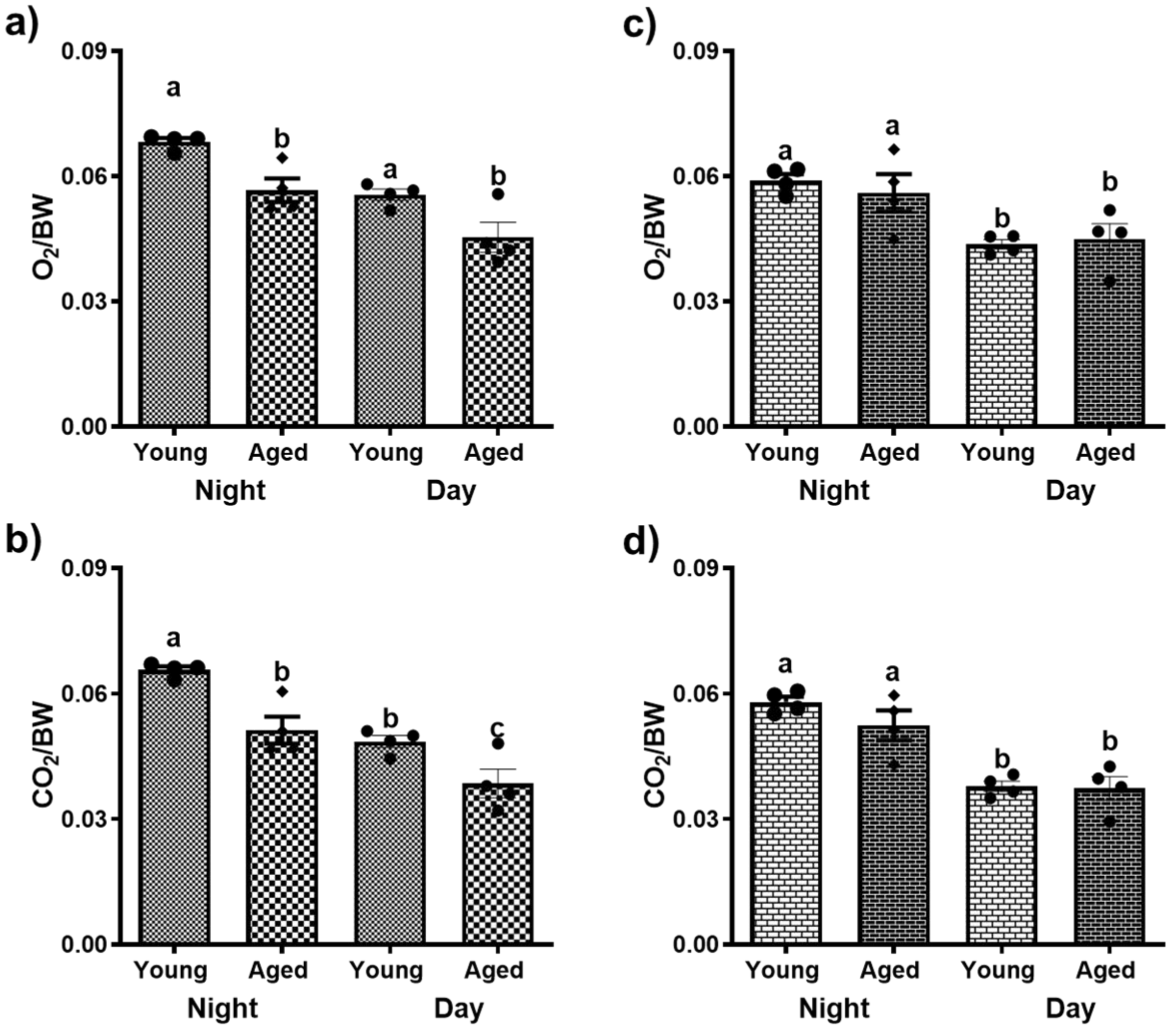

Figure S3. Measurement of metabolic rates of young and aged males (a, b) and females (c, d) mice estimated through (a, c) oxygen (O_2) consumption, (b, d) carbon dioxide (CO_2) production. Values are mean ± SEM (n = 5); values not sharing a common letter significantly differ from each other at $p \leq 0.05$.

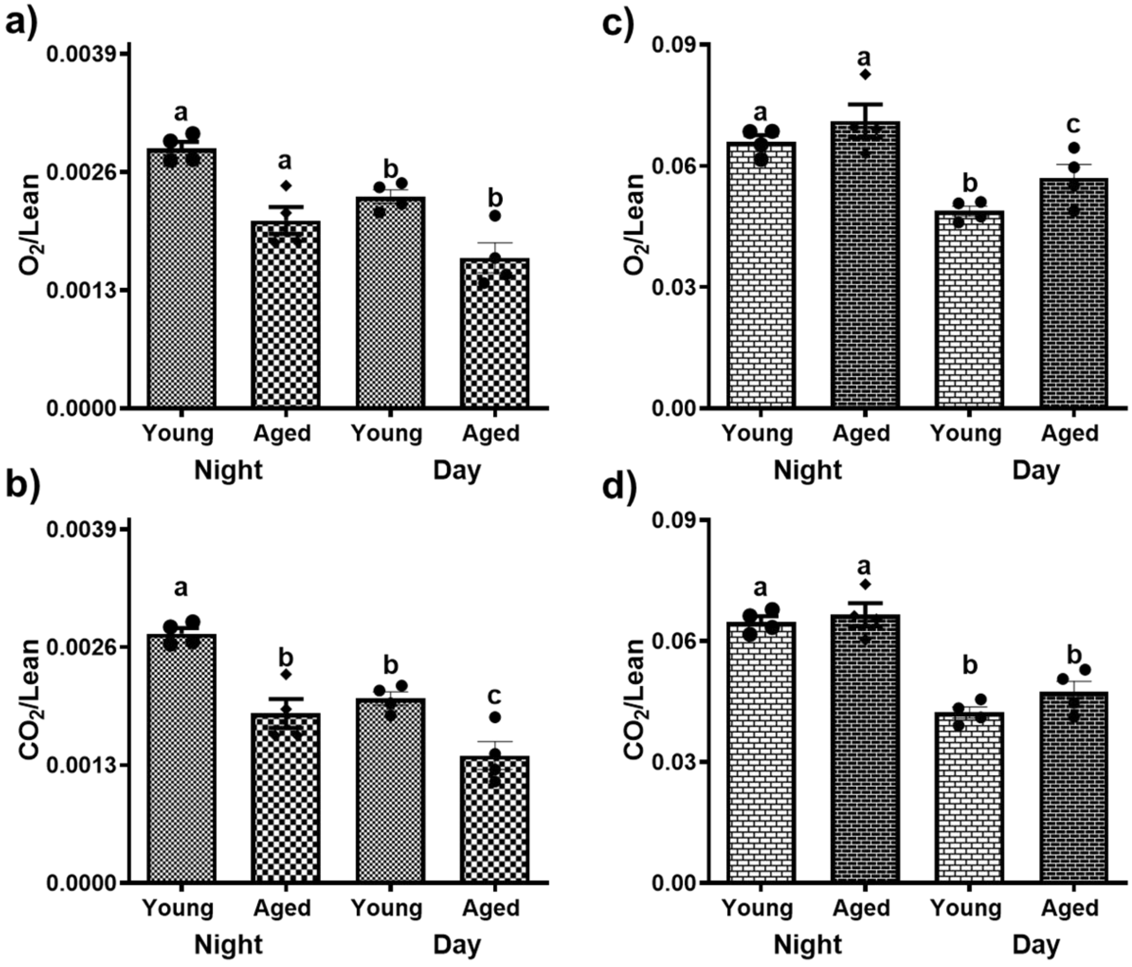

Figure S4. Measurement of metabolic rates of young and aged male (a, b) and female (c, d) mice normalized to lean mass. (a, c) oxygen (O_2) consumption, (b, d) carbon dioxide (CO_2) production. Values are mean ± SEM (n = 5); values not sharing a common letter significantly differ from each other at $p \leq 0.05$.

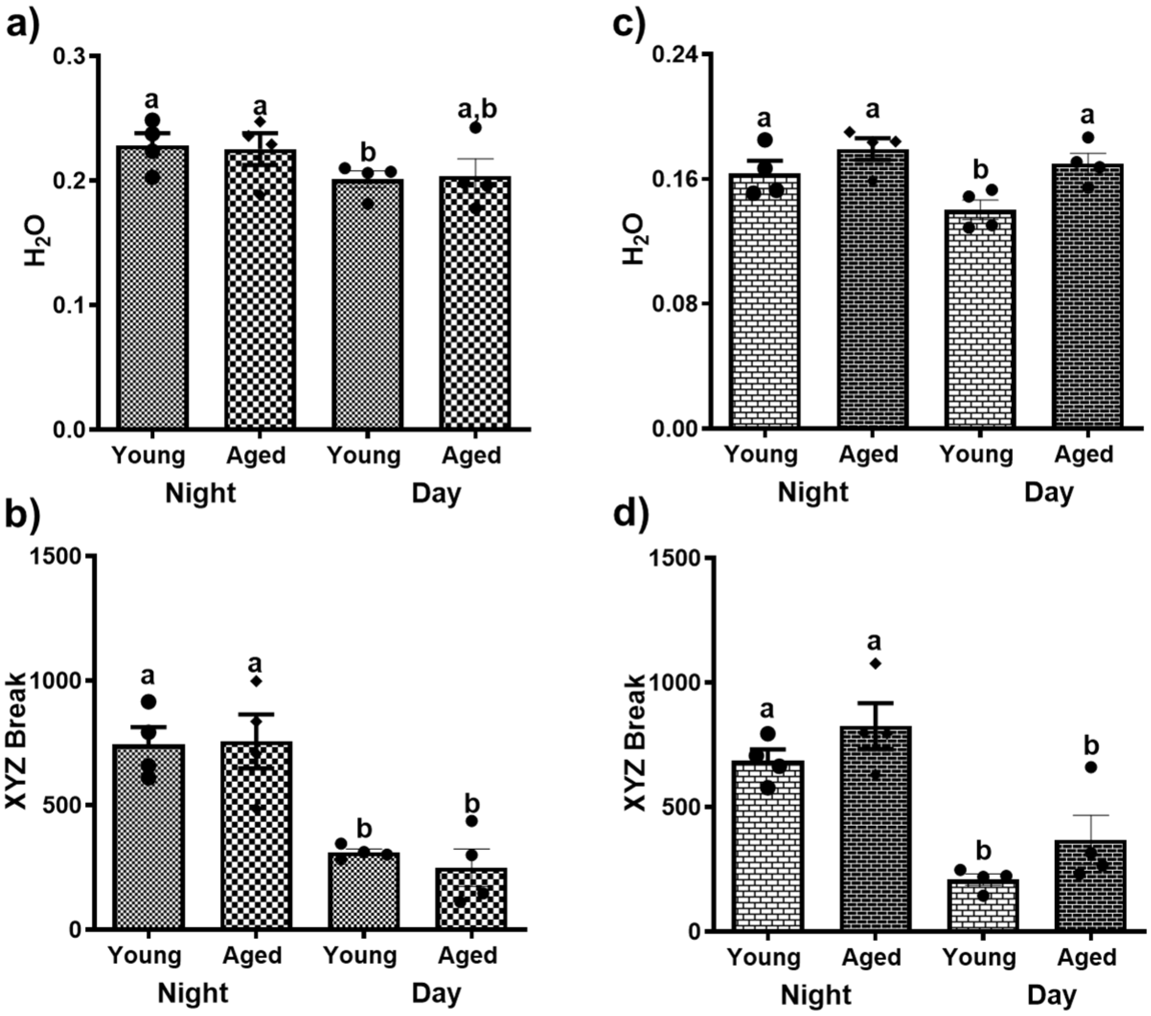

Figure S5. Measurement of metabolic water production in (a) males, and (c) females. XYZ breaks in (b) males and in (d) females. Values are mean ± SEM (n = 5); values not sharing a common letter significantly differ from each other at $p \leq 0.05$.

Supplemental Table 1. List of antibodies utilized in the study

Primary Antibody	Host	Cat. No.	Manufacturer
β-actin	Mouse	66009-1-Ig	Protein Tech
Malondialdehyde (MDA)	Mouse	MA5-27560	Invitrogen
4-Hydroxynonenal (4-HNE)	Mouse	MA5-27570	Invitrogen
p53/TRP53	Mouse	MA5-12453	Invitrogen
p21/CDKN1A	Rabbit	14-6715-81	Invitrogen
Tumor necrosis factor-α (TNF-α)	Mouse	6029-1-Ig	Protein Tech
Interleukin 1β (IL-1β)	Mouse	1242S	Cell Signaling Technology
CD68	Rabbit	Ab125212	Abcam
Myeloperoxidase (MPO)	Goat	PA5-143017	Invitrogen
Smooth muscle actin-α (α-SMA)	Mouse	A2547	Sigma
Collagen II	Mouse	NB600-488	Novus

Secondary Antibody	Host	Cat. No.	Manufacturer
Peroxidase AffiniPure Goat Anti-Rabbit IgG	Goat	111-035-144	Jackson ImmunoResearch laboratories
Peroxidase AffiniPure Goat Anti-Mouse IgG	Goat	115-035-166	Jackson ImmunoResearch laboratories
Donkey Anti-Goat IgG HRP	Donkey	V805A	Promega

Supplemental Table S2. List of TaqMan® FAM-labeled primers used for quantitative PCR

Gene Name	Assay ID	Manufacturer
Fatty acid synthase (FASN)	Mm00662319_m1	Applied Biosystems
Adipose tissue triglyceride lipase (ATGL/PNPLA2)	Mm00503040_m1	Applied Biosystems
Patatin-like phospholipase domain-containing protein 3 (PNPLA3)	Mm00504420_m1	Applied Biosystems
Tumor necrosis factor alpha (TNF-α)	Mm00443258_m1	Applied Biosystems
Interleukin 1 beta (IL-1β)	Mm00434228_m1	Applied Biosystems
Chemokine (C-C motif) ligand 2 (CCL2)	Mm00441242_m1	Applied Biosystems
Chemokine (C-X-C motif) ligand 2 (CXCL2)	Mm00436450_m1	Applied Biosystems
Transforming growth factor beta 1 (TGFβ1)	Mm01178820_m1	Applied Biosystems
Collagen, type I, alpha 1 (Col1α1)	Mm00801666_g1	Applied Biosystems
